# A building block for hardware belief networks

**DOI:** 10.1038/srep29893

**Published:** 2016-07-21

**Authors:** Behtash Behin-Aein, Vinh Diep, Supriyo Datta

**Affiliations:** 1 GLOBALFOUNDRIES Inc., Santa Clara, CA 95054, USA; 2 School of ECE, Purdue University, West Lafayette, IN 47907, USA

## Abstract

Belief networks represent a powerful approach to problems involving probabilistic inference, but much of the work in this area is software based utilizing standard deterministic hardware based on the transistor which provides the gain and directionality needed to interconnect billions of them into useful networks. This paper proposes a transistor like device that could provide an analogous building block for probabilistic networks. We present two proof-of-concept examples of belief networks, one reciprocal and one non-reciprocal, implemented using the proposed device which is simulated using experimentally benchmarked models.

Probabilistic computing is a thriving field of computer science and mathematics and is widely viewed as a powerful approach for tackling the daunting problems of searching, detection and inference posed by the ever increasing amount of “big data”^1^,^2^,^3^,^4^,^5^,^6^,^7^,^8^,^9^,^10^,^11^. Much of this work, however, is software-based, utilizing standard general purpose hardware that is based on high precision deterministic logic^12^. The building block for this standard hardware is the ubiquitous transistor which has the key properties of gain and directionality that allow billions of them to be interconnected to perform complex tasks. This paper proposes a transistor-like device that could provide an analogous building block for probabilistic logic.

A number of authors^13^,^14^,^15^ have recognized that the physics of nanomagnets can be exploited for stochastic logic and natural random number generators to replace the complex circuitry that is normally used. However, these are individual stochastic circuit elements within the standard framework of complementary metal oxide semiconductor (CMOS) transistors, which provide the necessary gain and directionality. By contrast, what we are proposing in this paper are networks constructed out of magnet-based stochastic devices that have been individually engineered to provide transistor-like gain and directionality so that they can be used to construct large scale circuits without external transistors (Fig. 1a).

Feynman (1982) alluded to a probabilistic computer based on probabilistic hardware that could efficiently solve problems involving classical probability, contrasting it with a quantum computer based on quantum hardware that could efficiently solve quantum problems. This paper inspired much work on quantum computing, but we would like to draw attention to his description of a probabilistic computer: “… *the other way to simulate a probabilistic nature*, *which I’ll call N* .. *is by a computer C which itself is probabilistic*, .. *in which the output is not a unique function of the input*. … *it simulates nature in this sense*: *that C goes from some* .. *initial state* .. *to some final state with the same probability that N goes from the corresponding initial state to the corresponding final state*. … *If you repeat the same experiment in the computer a large number of times* … *it will give the frequency of a given final state proportional to the number of times*, *with approximately the same rate* … *as it happens in nature*.” The possibility of probabilistic computing machines has also been addressed by more recent authors^16^,^17^,^18^,^19^,^20^,^21^,^22^. The primary purpose of this paper is to introduce the concept of a ‘transynapse’, a device that can be interconnected in large numbers to build probabilistic computers (Fig. 1).

The transynapse combines a synapse-like function with a transistor-like gain and directionality and in the next section we describe a device that uses the established physics of nanomagnets to implement it. We present a specific design for the transynapse which is simulated using experimentally benchmarked models (Supplementary section 1) for established phenomena to demonstrate the stochastic sigmoid transfer function. Reciprocal and non-reciprocal networks are discussed next where the same models are used to show how transynapses can be used to build either of two fundamentally different class of networks, an Ising like network with symmetric interactions and a non-Ising network with directed interactions. We then present two proof-of-concept examples of belief networks, one reciprocal for Boltzmann machines and the other non-reciprocal for Bayesian networks, implemented using transynapses.

## Transynapse: The Building Block

**Figure 1:**
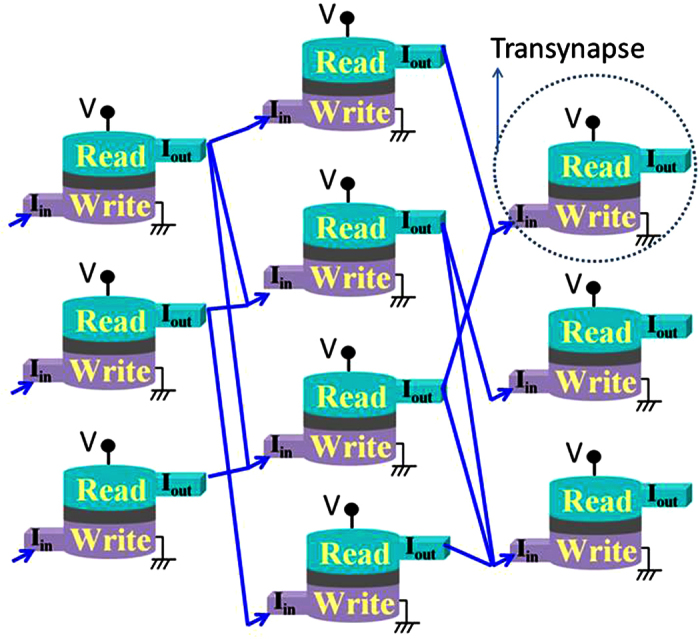
This paper defines a transynapse that can be interconnected to build probabilistic networks as shown schematically. Next section describes a specific transynapse design based on experimentally benchmarked models which are then used to illustrate the use of transynapse networks to solve problems involving Boltzmann machines and Bayesian networks.

**Figure 2:**
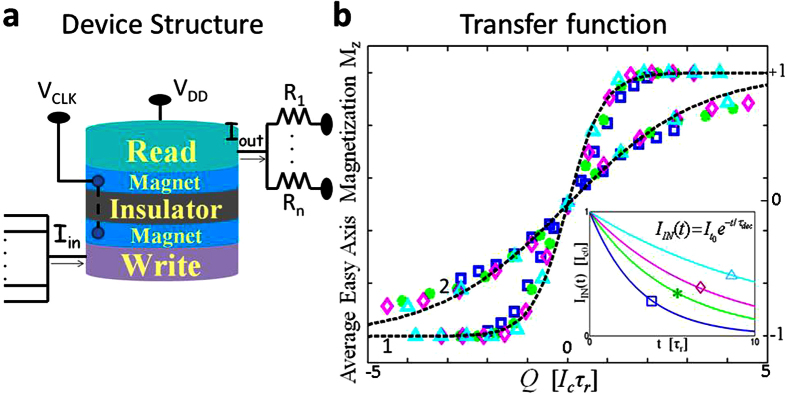
Design for a transynapse: (**a**) Device structure: For our simulations we use the same design as that in Datta *et al.*^23^ which provides the required gain, fan-in and fan-out, making use of the established physics of the spin Hall effect (SHE) for the input and the magnetic tunnel junction (MTJ) for the output. (see also refs 32,33) However, instead of the deterministic mode described earlier, we operate it in a probabilistic mode as described next. (**b**) The WRITE and READ magnets are both initialized along their hard axis and allowed to relax in the presence of an exponentially decaying current (see inset) *I*_*IN*_(*t*) and decay time parameter *τ*_*dec*_. The outputs obtained from a statistical average of 100 Monte Carlo runs for different input currents all fall on a single universal curve when plotted against *Q*, the time integrated current weighted by the factor 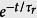. The dashed curves 1 and 2 are obtained for nanomagnets with energy barriers 48 *k*_*B*_*T* and 12 *k*_*B*_*T* respectively and are described well by Eq. 4.

The transynapse consists of a WRITE unit and a READ unit (Figs 1 and 2), electrically isolated from each other. The WRITE unit of transynapse *T*_*i*_ sums a set of input signals *I*_*IN*,*j*_(*t*), integrates them over a characteristic time scale *τ*_*r*_ to obtain a quantity


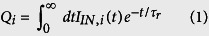


which determines the *mean value* of state *S* of the device through a sigmoidal function of the form


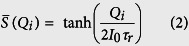


where *I*_0_ is a characteristic current. The READ unit produces multiple weighted output currents proportional to the average state 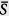:


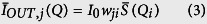


In this paper we use a specific design for this device following that described in Datta *et al.*^23^ which uses an input WRITE unit magnetically coupled to, but electrically isolated from an output READ unit. It provides the required gain, fan-in and fan-out, making use of experimentally benchmarked models (Supplementary section 1) for the established physics of the spin Hall effect (SHE) for the input and the magnetic tunnel junction (MTJ) for the output. However, for transynapse operation, we need to operate it in a probabilistic mode not considered before, where the input and output are not deterministic variables but stochastic ones. This could be done by using nanomagnets with low energy barriers (*E*_*b*_ < 5 *k*_*B*_*T*) that are in the super paramagnetic^24^ regime considering long enough programming times. The output (y-axis in Fig. 2b) could then be interpreted as the time averaged magnetization of a single magnet along its easy axis. However, in this paper we use a different approach as explained below.

For the simulations presented in this paper, nano-magnets are initialized along their hard axis at t = 0 and then allowed to relax. The output is obtained from a statistical average of the magnetization *M*_*z*_ along the easy axis obtained from 100 Monte Carlo runs based on the stochastic Landau-Lifshitz-Gilbert (LLG) equation, one for each magnet (WRITE and READ) coupled through a magnetic interaction as in ref. 23. The details are described in the methods section. Figure 2b shows that the numerically obtained average *M*_*z*_ is described well by the relation


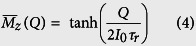


where *τ*_*r*_ = *f*_*T*_(1 + *α*^2^)/(2*αγH*_*k*_), *H*_*K*_ being the anisotropy field, *γ*, the gyromagnetic ratio and *α* is the damping parameter. Also 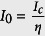, where *I*_*c*_ is the switching current for the nanomagnet^25^, while the factor *η* depends on its energy barrier *E*_*b*_ and is given by the relation *η* ≈ 0.06(*E*_*b*_/*k*_*B*_*T*)^0.94^ obtained from numerical simulations. Factor *f*_*T*_ (6 for *E*_*b*_ = 48 *k*_*B*_*T* and 24 for *E*_*b*_ = 12 *k*_*B*_*T*) determines how fast the magnetization relaxes depending on the ambient temperature. The results were obtained using different (Fig. 2b inset) input currents of the form 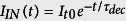, but the resulting output is well described by a single curve *M*_*z*_(*Q*) irrespective of the amplitude *I*_*t*0_ and decay time parameter *τ*_*dec*_. This independence to time-decay parameters suggests that the probability of finalizing a magnet in one of the two states essentially depends on the number (*N*_*s*_ where *Q* ∝ *N*_*s*_) of Bohr magnetons (units of electron spin) imparted on it^26^. Indeed, similar underlying principles have been demonstrated experimentally in ref. 27 in somewhat different set ups for switching magnets from one state to the other in the short pulse regimes well above *I*_*c*_.

It is important to note the key attributes of the device that are needed to enable the construction of belief networks by interconnecting hundreds of devices. Firstly, it is important to ensure input-output isolation, which is achieved by having magnetically coupled WRITE and READ magnets separated by an insulator as shown. This separation would not be needed if the magnet itself were insulating (like YIG, Yttrium iron garnet). The second important attribute is its gain defined as the maximum output charge current relative to the minimum charge current needed to swing the probability from 0.5 (fully stochastic) to 1 (fully deterministic). This is the quantity that determines the maximum fan-out that is possible which is particularly important if we want a high degree of inter connectivity. The physics of SHE^28^,^29^,^30^,^31^ helps provide gain since for each device, it can be designed^23^,^32^,^33^ to provide more spin current to the WRITE magnet than the charge current provided by the READ unit of the preceding stage.

The third attribute of the proposed device is its ability to sum multiple inputs and this can be done conveniently since it is current-driven. A WRITE circuit consisting of a SHE metal like Tantalum provides a current-driven low impedance input, different from the voltage-driven high input impedance field-effect transistors (FET’s). The low input impedance ensures that the total current into the WRITE unit is determined by the output impedance of the READ units of preceding stages^23^,^32^,^33^. This impedance is set by the intrinsic resistance of the READ units which could be on the order of a *k*Ω if using magnetic tunnel junctions (MTJ’s) or could be much lower if using the inverse spin Hall effect (ISHE)^34^. In either case an external series resistor R could be used to raise the output impedance as shown in Fig. 2a.


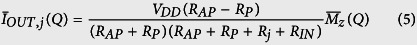


*V*_*DD*_ being the external voltage, *R*_*P*_ and *R*_*AP*_, the parallel and anti-parallel resistance of the MTJ, *R*_*IN*_, the input resistance of the next device and *R*_*j*_ is the external series resistance which can be used to weight the outputs appropriately. The weighting of the output can also be accomplished by tuning *V*_*DD*_ where multiple bipolar output weights sharing the same input can be implemented via a common WRITE unit with multiple READ units (An example of this is shown in the Bayesian network section)^35^.

We envision that the detailed physics used to implement the transynapse will evolve, especially the physics used for the WRITE, the READ and/or the weighting, since this field is in a stage of rapid development with new discoveries being reported on a regular basis. The input (or WRITE) circuit could utilize phenomena other than the SHE used here, just as the output (or READ) circuit could use mechanisms other than MTJ’s. Similarly, the nanomagnet can be initialized in a neutral state with modern voltage driven mechanisms^36^,^37^ like voltage controlled anisotropy, or with established methods like an external magnetic field^38^,^39^ or spin torque^30^,^40^,^41^, or thermal assistance^42^. Alternatively, as mentioned earlier nanomagnets in the super paramagnetic^24^ regime could be used with the mean state 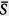 defined by a time average instead of an ensemble average. The purpose of this manuscript is simply to establish the general concept of a transynapse that integrates a synapse-like behavior with a transistor-like gain and isolation, thus permitting the construction of compact large scale belief networks.

Note also that our transynapses are assumed to communicate via charge current since that is a well-established robust form of communication. However, communication could be influenced through spin channels (as in all-spin logic^40^,^43^) or through spin waves requiring very different WRITE and READ units.

## Reciprocal and Non-Reciprocal Networks

A key feature of transynapse is the flexibility it affords in adjusting the weight *w*_*ji*_ that determines the influence of one transynapse (*T*_*i*_) on another (*T*_*j*_), by adjusting the parameters of the READ unit of *T*_*i*_. The weight *w*_*ij*_ on the other hand is controlled independently through the READ unit of *T*_*j*_. If we choose *w*_*ij*_ = *w*_*ji*_, we have a bidirectional or reciprocal network similar to the type described by an Ising model described by a Hamiltonian *H*. In such networks the probability *P*_*n*_ of a specific configuration, 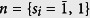 is known to be given by the principles of equilibrium statistical mechanics.


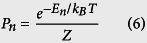


where the energy *E*_*n*_ of configuration *n* is given by


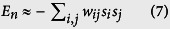


Ising models are closely related to Boltzmann machines^1^,^2^,^5^,^6^,^10^,^11^ whose probabilities described by Eq. 6 seek configuration with low *E*_*n*_. For example, with three transynapses connected through *w*_*ij*_ = *w*_*ji*_ > 0, *E*_*n*_ is minimized for configurations (111) and 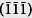 with equal *s*_*i*_. This is the ferromagnetic (FM) Ising model. But if *w*_*ij*_ = *w*_*ji*_ < 0, *E*_*n*_ would be a minimum if all *s*_*i*_ had opposite signs. Since this is impossible with three transynapses, the energy is lowest for all six configurations that have one ‘frustrated’ pair^44^,^45^:


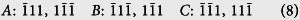


**Figure 3:**
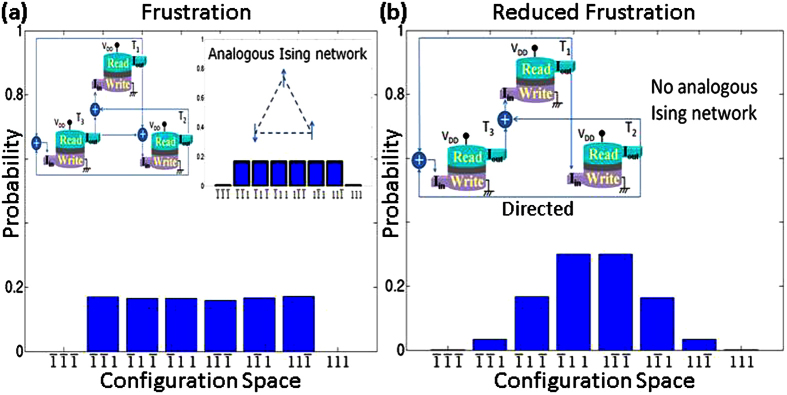
(**a**) Three Transynapses are initialized and then left to relax while interacting in a pairwise manner. The strength of interactions depends on voltage *V*_*DD*_. The polarity of *V*_*DD*_ for each transynapse is such that it favors the next transynapse to have an opposite state to its own as in anti-ferromagnetic (AF) ordering. Statistical information is then gathered from Monte-Carlo runs. There are a total of 2^3^ configurations possible with their probabilities shown in (**a**). This is reminiscent of frustration in spin glasses^44^,^45^ also observed in Ising model as shown in the inset. Such bidirectional connections can be used for building Boltzmann machines^10^,^11^ closely related to Ising models. More on this in Fig. 4. (**b**) Changing one of the connections in part (**a**) to be directed as opposed to bi- directional lowers the probability of occurrence of some states in the final configuration resulting in reduced frustration. This is fundamentally not possible by inherently symmetric Hamiltonian based systems such as Ising model. Such directed connections can be used to represent causal influences in Bayesian networks^3^,^4^. More on this in Figs 5 and 6.

The numerical simulation of the 3-transynapse network shows (Fig. 3a) this expected behavior with equal probabilities for configurations A, B, C, and reduced probabilities for the two remaining configurations (111) and 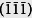 for which all three pairs are frustrated. Situation is different when one of the bonds is directed as in Fig. 3b. Not surprisingly, the probability is highest for the configuration having *T*_2_ and *T*_3_ as the frustrated pair (configuration A in Eq. 8). Less obviously, configuration B with *T*_1_, *T*_3_ as the frustrated pair has a higher probability than configuration C with *T*_1_, *T*_2_ as the frustrated pair. This is because *T*_2_ only has one bond (from *T*_1_) dictating its state (no conflict) but *T*_3_ has two bonds (from *T*_1_ and *T*_2_) dictating its state which can be at odds with each other. Such configuration of bonds and the resulting configuration space probabilities as shown in Fig. 3b have no Ising analog.

Note that our numerical results are all obtained directly by simulating a set of coupled LLG equations, one for each of the six magnets, two per transynapse. The time evolution of each magnet in each device is a function of its instantaneous state 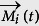, internal, external and thermally fluctuating fields (determined by temperature *T*), plus the spin torque 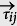 it receives from other devices:


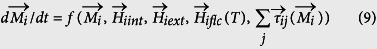


Bi-directional interactions have both 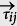 and 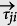 but directional interactions have either 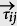 or 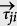. Methods section provides more detail.

## Implementing Belief Networks

### Boltzmann machine

**Figure 4:**
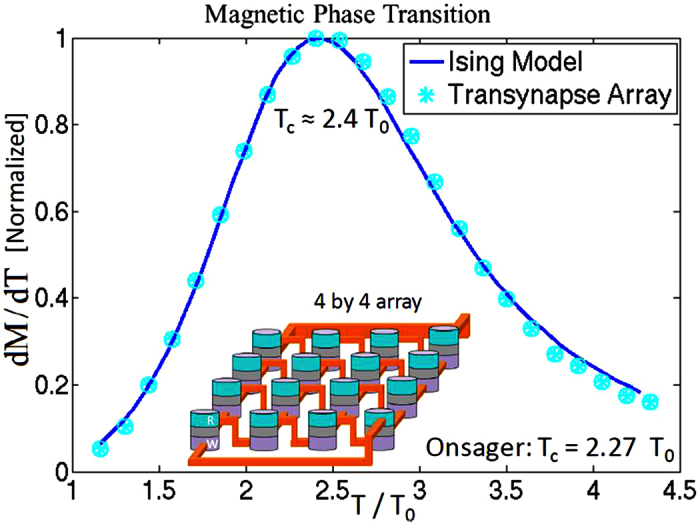
A 4 by 4 array of transynapses with nearest neighbor connections (The inset is intended to illustrate these aspects and not the directionality of connections). Same *V*_*DD*_ (*V*_*DD*_ < 0) is applied to all transynapses making the interactions favor all devices in the same state similar to ferro-magentic ordering (FM). At each temperature *T* (scaled by *T*_0_ ≡ *J*/*k*_*B*_, where *J* is the coupling strength. See also Supplementary sections 3 and 4), the circuit is initialized and left to interact while the network decides on a final state out of 2^16^ possible states. After each trial, the magnetization of the array is obtained by summing over all transynapse states leading to an average magnetization of the array based on the total number of Monte Carlo runs. From this data, differential of magnetization with respect to temperature can be obtained as shown. This is reminiscent of magnetic phase transition^49^ exhibiting a Curie temperature in Ising model depicted by the solid line. In deep belief networks^1^,^2^,^8^,^9^, the closely related restricted Boltzmann machines^10^,^11^ trained by breakthrough algorithms that determine the interactions are used to solve search, detection and inference problems^5^,^7^,^8^.

The connection between Ising model of statistical mechanics^46^ and hard combinatorial optimization problems of mathematics has been known for decades^47^. Boltzmann machines^10^,^11^ and subsequently their restricted version for deep belief networks are Ising models in which the weights of interactions are learned and adjusted with breakthrough algorithms^1^,^2^,^5^. There is also widespread activity and innovation on the connection of inference, commonly used in belief networks, and phase transitions in statistical physics (see e.g. ref. 48 for a thorough review). Figure 4 shows how networks described in this paper (Fig. 1) can mimic magnetic phase transition which is also a well known result of the Ising model. The caption provides more detail for the particular procedure used for obtaining this. Phase transition is evident as the rate of change of magnetization with respect to temperature exhibits a maximum followed by a decrease. This transition is not sharp because of the small lattice sizes used here (see ref. 49 for a more in-depth discussion). Solid line shows the analogous Ising model result with the same lattice size (4 by 4 array) using equilibrium laws of statistical mechanics. The peak exhibited is reminiscent of the Curie temperature of magnetic phase transition (Supplementary section 3). Indeed, the effective Curie temperature observed in these networks depends linearly on the strength of device to device communication set by *V*_*DD*_ (Fig. 2). (Supplementary section 4 provides spontaneous magnetization curves with interactions of various strength). This is in agreement with Onsager’s^50^ results for a two dimensional array of ferromagnetic atoms for which *T*_*C*_ is proportional to *J*-coupling strength. We take these as an indication that stochastic networks of transynapses could be used to construct (restricted) Boltzmann machines for deep belief networks where weights can be adjusted by the bipolar voltages applied to transypases or by load resistances at the output of transynapses (Fig. 2).

### Bayesian network

**Figure 5:**
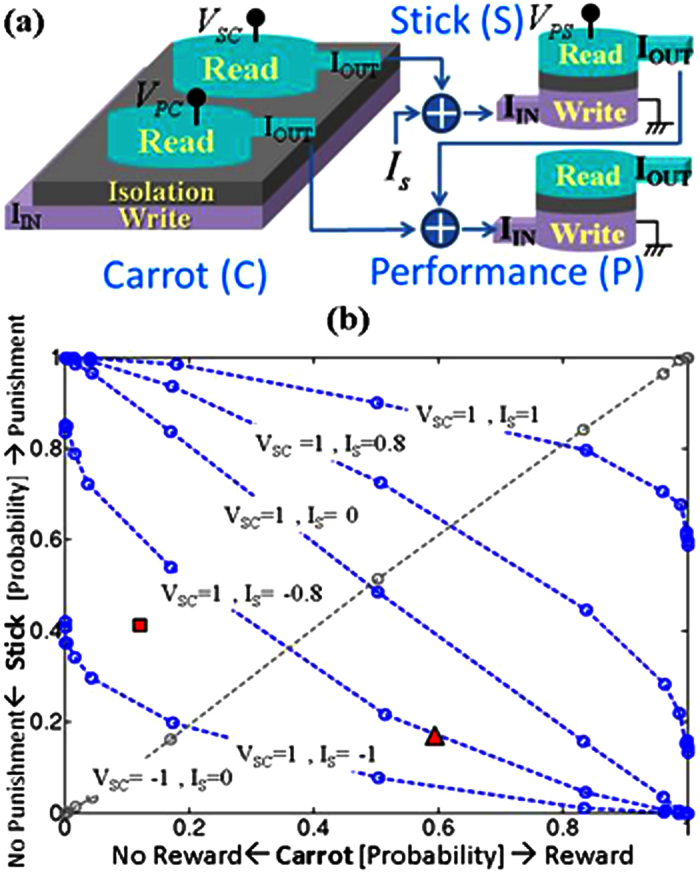
(**a**) Directed circuits of Transynapses (Fig. 1) can represent Bayesian networks in which directionality can represent causality^3^,^4^. Unlike Hamiltonian systems (e.g. Ising model), the interactions are not symmetric. Here, carrot influences both the state of the stick and the performance while stick also affects performance. (**b**) Direct simulation of (**a**). Figure shows a probabilistic gate in which diagonal lines represent perfect correlation of stick with carrot (probabilistic COPY) using *V*_*SC*_ = −1 and perfect anti-correlation (probabilistic NOT) using *V*_*SC*_ = +1. (voltages and currents are normalized by the magnitude required for deterministic switching). When *I*_*S*_ ≠ 0, statistical correlation varies e.g. the stick can be in the no-punishment mode irrespective of the carrot.

Symmetric interactions are inherent to Hamiltonian based systems as in Ising model and Boltzmann machines. On the other hand, directed interactions have their own prominence in Bayesian networks^3^,^4^. Figure 5a shows a 3-transynapse network, with each transynapse representing one of three variables which we could call carrot, stick and performance as shown in Table 1. These variables can be in one of two possible states with distinct probabilities. The transynapse network is interconnected to reflect the causal interconnections among the three variables. The carrot affects both the state of stick and the state of performance through the voltages *V*_*SC*_ and *V*_*PC*_ which determine the weights *w*_*SC*_ and *w*_*PC*_. The only other causal effect is that of the stick on the performance which is reflected in the voltage *V*_*PS*_ and the resulting weight *w*_*PS*_.

A direct simulation of this 3-transynapse network using coupled LLG equations (Eq. 10) yields the plot shown in Fig. 5b. With *V*_*SC*_ = −1, we get the diagonal lines reflecting perfect correlation of the stick with the carrot, while with *V*_*SC*_ = +1, we get the other diagonal line reflecting perfect anti-correlation. (Voltages and currents are normalized by the magnitude required for deterministic switching). We could view these respectively as a COPY gate and a NOT gate with probabilistic inputs and outputs. The other curves shown in Fig. 5b correspond to *I*_*S*_ ≠ 0 reflecting a situation where the stick state is not entirely controlled by the carrot, but has a probability of no punishment irrespective of the carrot.

The network can naturally generate probabilities of various variables. Consider e.g. the triangle (scenario A) in Fig. 5b where carrot has 0.6 probability of reward (scenario B is the square). Instead of performing the necessary algebra of 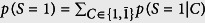 to obtain the probability of stick being in the punishment mode, the transynapse network takes in *I*_*C*_ = −0.02, *V*_*SC*_ = 1, *I*_*S*_ = 0.9 and produces the directly observable probability of stick being in punishment mode. (Voltages and currents are normalized by the magnitude required for deterministic switching). This generalizes to more variables and an example for three is discussed next.

**Figure 6:**
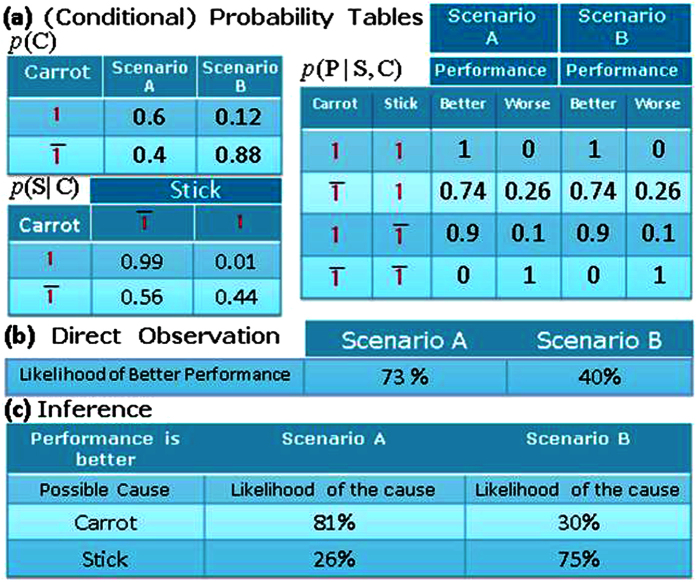
(**a**) (Conditional) probability tables: Two scenarios ((A) triangle and (B) square in Fig. 5b) are considered for the state of carrot. Such scenarios are typically provided by the problem statement which determines the voltages and currents applied to transynapses based on their transfer function (Fig. 2b). They in turn ensure that the network generates the probability values as shown. *V*_*SC*_ = 1, *V*_*PC*_ = −0.4, *V*_*PS*_ = −0.5, *I*_*C*_ = −0.02, *I*_*S*_ = 0.9, *I*_*P*_ = −0.2 are used for scenario A. Same values are used for scenario B except that *I*_*C*_ = 0.1. (voltages and currents are normalized by the magnitude required for deterministic switching). (**b**) Likelihood of better performance can be directly observed without using (**a**) and carrying out the algebra for *p*(*P* = 1|*S*, *C*). (**c**) Inference can be addressed by such networks. For example, the likelihood that reward has caused better performance is the mean value of carrot in the reward state for cases that have better performance.

Figure 6a,b shows how the network in Fig. 5a can be used in predictive mode based on known casual connections^3^,^4^ among different variables which determine the electrical signals *V*_*ij*_ and *I*_*i*_ (explicitly provided in the caption). These in turn can provide the values in the (conditional) probability tables of Fig. 6a. For example, the element indexed by 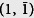 in the *p*(*S*|*C*) table is the mean value of the state of stick in the 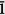 mode when *C* = 1. (This can also be obtained independently by dictating the carrot is in the reward (*C* = 1) state e.g. by providing a strong bias (*I*_*C*_) and finding the mean value for the state of stick due to *V*_*SC*_) While the likelihood of better performance can be found from tables of Fig. 6a by calculating *p*(*P* = 1|, *S*, *C*), this is directly observable from the mean value of the state of ‘performance’ which is naturally generated by the network as provided in Fig. 6b. Alternatively, the network can address inference problems. Suppose performance is better, is it due to carrot or stick or both? For instance, the likelihood that performance is better because of reward is essentially *p*(*C* = 1|*P* = 1). This can be obtained by the algebra, 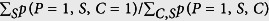, or directly observed by taking the mean value of the state of carrot in the reward mode when performance is better. The resulting values are provided in Fig. 6c.

## Concluding Remarks

Probabilistic computing is a thriving field of computer science and mathematics that deals with extracting knowledge from available data to guide decisive action. The work in this area is largely based on deterministic hardware and major advances can be expected if one could build probabilistic hardware to simulate probabilistic logic. In this paper we define a building block for such stochastic networks, which we call a transynapse combining the transistor-like properties of gain and isolation with synaptic properties. In principle, many implementations are possible including those that make at least some use of standard CMOS circuitry. We present a possible implementation based on the established physics of nano magnets for the transynapse and use experimentally benchmarked models to illustrate the implementation of both Boltzmann machines and Bayesian networks. More realistic examples will be addressed in future publications^51^.

## Methods and Verification

This section outlines the methodology and its implementation underlying the simulations that have been carried out. It includes the steps taken to verify the model against well known principles or experimental data.

The time evolution and final state of each nano-magnet 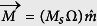 is represented and simulated by the Landau-Lifshitz-Gilbert (LLG) equation:


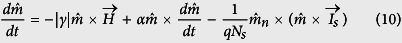


where *q* is the charge of electron, *γ* is the gyromagnetic ratio, *α* is the Gilbert damping coefficient and *N*_*s*_ ≡ *M*_*s*_Ω/*μ*_*B*_ (*M*_*s*_: saturation magnetization, Ω: volume) is the net number of Bohr magnetons comprising the nanomagnet and 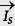 is the spin current entering the magnet.

This equation is transformed to its standard mathematical form (see e.g. ref. 40) and is solved numerically using a second order Runge-Kutta method (a.k.a Heun’s method) in MATLAB. This methodology essentially applies the Stratonovich stochastic calculus to the stochastic integration during time dependent simulations involving thermal fluctuations. The inclusion of thermal fluctuations in LLG and its implementation has been verified against equilibrium laws of statistical mechanics in ref. 40.


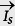
, the spin current entering the magnet, can have components both due to the Slonczweski torque as well as the field-like torque. The inclusion of spin transfer torque in LLG (last term) and its implementation has been verified against experimental data in ref. 26. In this manuscript, 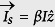 is generated by the spin Hall effect as outlined in ref. 23 where *I* is the charge current entering the *W* unit generated by the READ stage of the previous device (see Figs 1, 2, 3)^23^. This is essentially how Transynapses communicated with each other whereby one drives the next; the dynamics of each one being governed by the coupled LLG equations that describe the dynamics of READ and WRITE magnets.

The magnetic field, 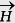, represents both internal and external fields:


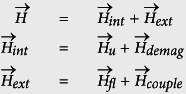


where 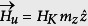 is the uniaxial anisotropy field with *z* as the easy axis and 
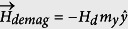
 is the demagnetizing field with *y* as the out of plane hard axis for in-plane magnets. *H*_*d*_ is zero for perpendicular anisotropy magnets.

The thermal fluctuating field, 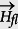, has the following statistical properties:


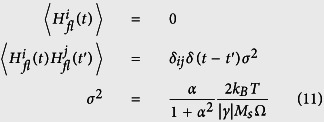


where *δ*(*t*) is the Dirac delta function, *δ*_*ij*_ is the Kronecker delta, and indices *i* and *j* are labels for the field’s vector components. *T* is temperature and *k*_*B*_ is the Boltzmann constant.

The coupling field, 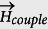, accounts for the magnetic interaction of the READ (R) and WRITE (W) magnets within each device as introduced and described in ref. 23 and illustrated in Fig. 2a of the main manuscript (There is no magnetic coupling envisioned between various devices here. Device to device communication happens via charge currents as described earlier.). This reference describes functionality of the spin switch and the governing equations and presents the coupled LLG equations describing the time dynamics of R and W magnets. The modeling of magnetic coupling between READ and WRITE magnets for in-plane magnetic materials has been described in detail in ref. 52 and verified against experimental data. Here, we review a brief description of how this coupling is calculated for perpendicular magnetic materials (PMA) along with the validation of its implementation against experimental data shown in the first figure of Supplementary information. For this, we follow the methodology described in ref. 53. Within each device, W and R magnets exert a magnetic field on the other. For example, the field exerted on the READ magnet from the WRITE magnet is 
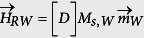
 where [D] is a 3 by 3 tensor describing the effect of each elemental volume of W magnet on each elemental volume of the R magnet integrated over the volume of both


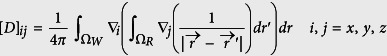


To validate the approach and its implementation, we made use of the available experimental data for the coupling fields that have been measured in magnetic tunnel junctions. First figure of the Supplementary Information shows the comparisons between the calculated values from the model and the data from various experiments. These comparisons show that the model is generally in good agreement with experimental demonstrations.

## Additional Information

**How to cite this article**: Behin-Aein, B. *et al.* A building block for hardware belief networks. *Sci. Rep.* **6**, 29893; doi: 10.1038/srep29893 (2016).

## Supplementary Material

Supplementary Information

## Figures and Tables

**Table 1:** Transynapses can represent nodes in Bayesian networks.

	Carrot (C)	Stick (S)	Performance (P)
1	Reward	Punishment	Better
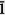	No Reward	No Punishment	Worse

See also Fig. 5.
