# Supplementary material for: A building block for hardware belief networks
Source: Sci Rep. 2016 Jul 21;6:29893. doi: 10.1038/srep29893 (PMC4956763; doi:10.1038/srep29893)
Supplement: Supplementary Information [file srep29893-s1.pdf]

Supplementary Information
A building block for hardware belief networks

Behtash Behin-Aein[1], Vinh Diep[2], and Supriyo Datta[2]

[1]GLOBALFOUNDRIES Inc. USA, Santa Clara, CA 95054
[2]School of ECE, Purdue University, West Lafayette, IN 47907

June 15, 2016

Abstract

Belief networks represent a powerful approach to problems involving probabilistic inference, but much of the work in this area is software based utilizing standard deterministic hardware based on the transistor which provides the gain and directionality needed to interconnect billions of them into useful networks. This paper proposes a transistor like device that could provide an analogous building block for probabilistic networks. We present two proof of concept examples of belief networks, one reciprocal and one non-reciprocal, implemented using the proposed device which is simulated using experimentally benchmarked models.

Contents

1 Coupling fields: Verification against experiments **2**

2 Model specifications and parameters **3**

3 Ising Model **3**

4 V_{DD} tuning of spontaneous magnetization and control of effective Curie temperature T_c **4**

1 Coupling fields: Verification against experiments

The method section described the approach used for calculating the coupling fields. Here the validation of approach against experimental data is shown in Fig.1.

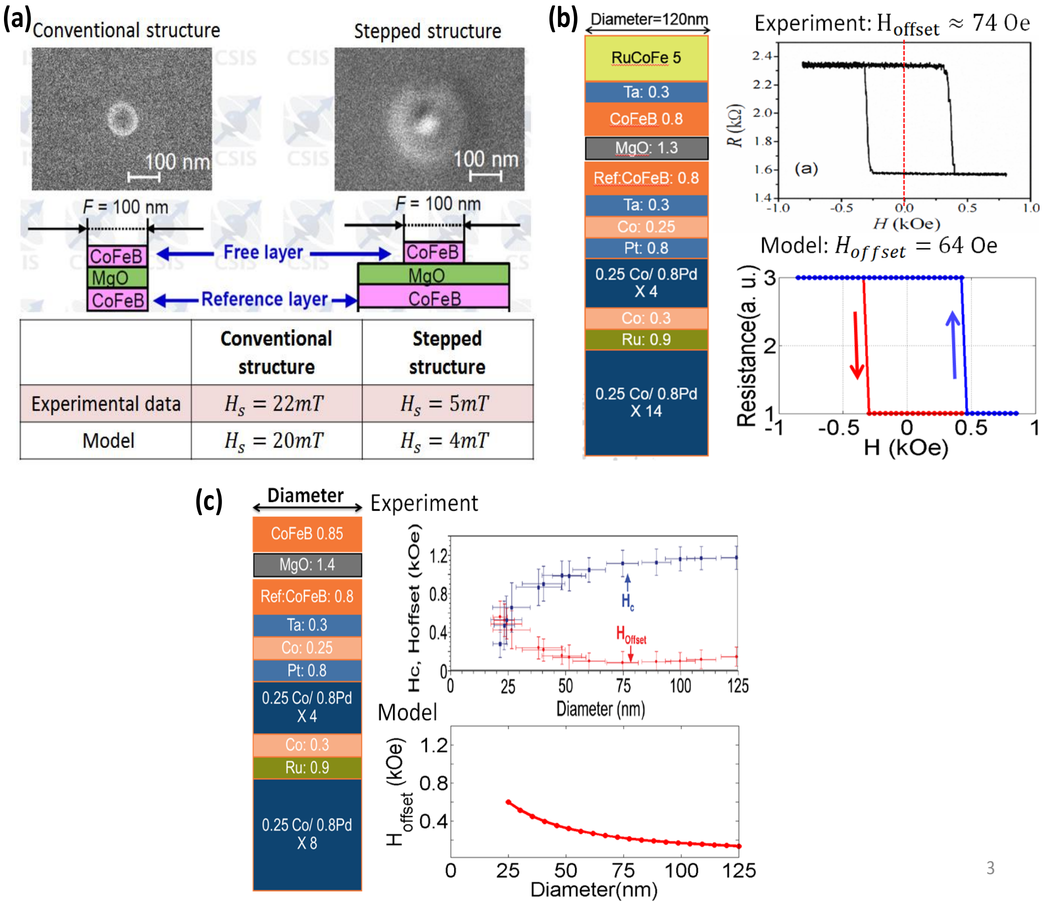

	Conventional structure	Stepped structure
Experimental data	$H_s = 22mT$	$H_s = 5mT$
Model	$H_s = 20mT$	$H_s = 4mT$

Figure 1: **(a)** Model is validated against reference [1] for the effect of the stray fields in shifting the R-H hysteresis loops for two different structures as described in the reference. **(b)** Model is validated against reference [2] for the effect of the stray fields in shifting the R-H hysteresis loops in MTJ structure as illustrated layer by layer on the left. **(c)** Model is validated against reference [3] for the scaling of coupling fields as the diameter of magnetic tunnel junctions are scaled.

2 Model specifications and parameters

The spin switch consists of various layers as discussed in Ref. [?]. The free coupled magnetic layers are nominally identical. Their dimensions are $100 * 100 * 2nm^2$ with $M_s = 1000$ emu/cc and $H_K = 200$ Oe for the $E_b \approx 48kT$ magnet and $H_K = 50$ Oe $E_b \approx 12kT$ magnet in Fig.2. Both have damping parameter of $\alpha = 0.01$. Both perpendicular anisotropy magnets and in-plane magnets can produce the stochastic sigmoid function like Fig.2. Figures 3,5 and 6 use lower energy barrier magnets which due to lower volume (100*50*2 nm^3) and anisotropy field ($H_K = 25$ Oe). The separation between these coupled magnets is 7 nm. Figure 4 which shows the magnetic phase transition uses magnets with very small barriers. This is to be more consistent with Ising spins which actually do not have any intrinsic energy barriers. The free layer specifications used in the transynapse network of figure 4 are 50*50*2 $nm3$ and $H_K = 20$ Oe. The "J-coupling" for the transynapse network was adjusted by V_{DD} to achieve a current that is twice the switching current of the each transynapse. This is the maximum magnitude of the current I_{cpl} that each transynapse sends to other ones. The connection between the J-coupling of the Ising model (discussed next) and the transynapse network was made by setting the J of transynapse network to to $I_{cpl}/4$.

3 Ising Model

The Ising model is a well-known established mathematical model of ferromagnetism in statistical mechanics. Where applicable, in the main manuscript, it has been used to draw comparison between networks of Ising spins and magnetic networks presented in this manuscript. For that purpose, a MATLAB script was written based on the governing equations outlined below. The standard form of this model describes the interaction energy or the Hamiltonian of a network of Ising spins which can be under the influence of an external magnetic field. For a network in state S, the many-body Hamiltonian of size 2^n by 2^n where n is the number of Ising spins can be written as

$$H_S = -\Sigma_{ij} J_{ij} S_i S_j - \mu \Sigma_i h_i S_i \qquad S_{i,j} \in \{+1, -1\}$$

Where J_{ij} describes the interaction energy between two nearest neighbor Ising spins S_i and S_j. Magnetic moment of each spin is denoted by μ and could be under the influence of an external magnetic field h_i. Depending on the values assumed by the Ising spins, the network can have various possible configurations (states). The probability of each state S at equilibrium in the configuration space is given by

$$P_S = \frac{e^{-\beta H_S}}{Z} \qquad \beta \equiv (k_B T)^{-1}$$

k_B is the Boltzmann constant and T is the ambient temperature. Z is the well-known partition function of equilibrium statistical mechanics $Z = \Sigma_S e^{-\beta H_S}$. Note that $\Sigma_s P_s = 1$ always.
Energy and magnetization of such a system are observable and can be readily found by taking the their expectation value

$$E = \Sigma_S H_S P_S$$

$$M = \Sigma_S M_S P_S$$

Here, M_s is the normalized net magnetization of each state of the many-body system found by summing over all Ising spin values of each state. For magnetic phase transition, we look at the heat

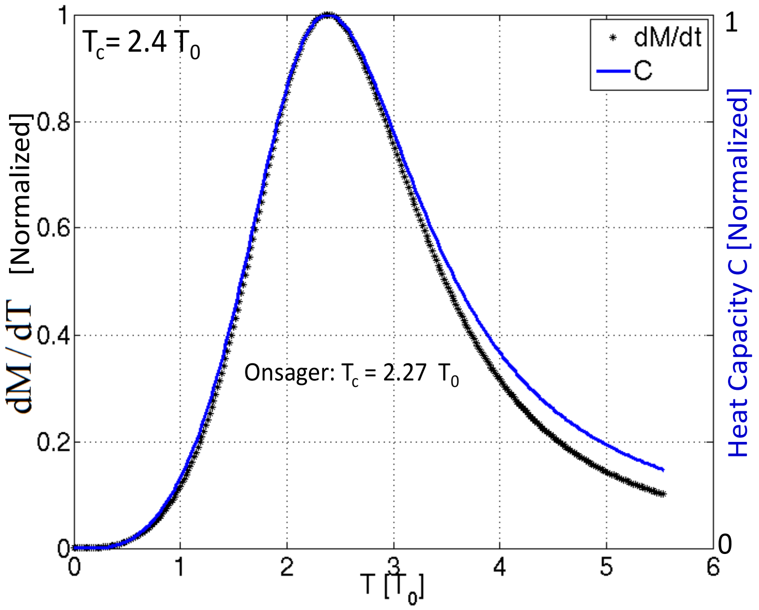

Figure 2: Heat capacity and rate of change of magnetization (phase) with respect to temperature (order parameter) plotted as a functional of temperature. Phase transition and Curie temperature are evident from both curves.

capacity as a function of temperature. Heat capacity, C, is a measure of how much heat is added to a system for a given temperature change

$$C = \frac{dE}{dT}$$

Curie temperature, T_C is the critical point at which the heat capacity (C) peaks while the material's phase (magnetization here) exhibits an inflection point; hence the phase change. It is easily recognizable in the C versus T plot (supplementary Fig.2). But it also is recognizable from the rate of change of materials' phase (magnetization) with respect to the order parameter (temperature here). This is discussed more in detail in the next section.

4 V_{DD} tuning of spontaneous magnetization and control of effective Curie temperature T_c

Supplementary Fig.3 shows spontaneous magnetization as a function of temperature for a transynapse network and Ising model (both are 4 by 4 arrays). The inflection point where the magnetization changes its curvature marks the Curie temperature point T_C which is the point at which the rate of change of magnetization peaks (supplementary Fig.2). What Fig.3 shows is that for a linear increase in V_{DD}, spontaneous magnetization curves are shifted linearly. This is in agreement with Onsager's derivation, $T_C = 2.27J/k_B$, essentially explaining the linear dependence of T_C on the coupling strength J between the Ising spins. To clarify this further, the inset shows an analogous

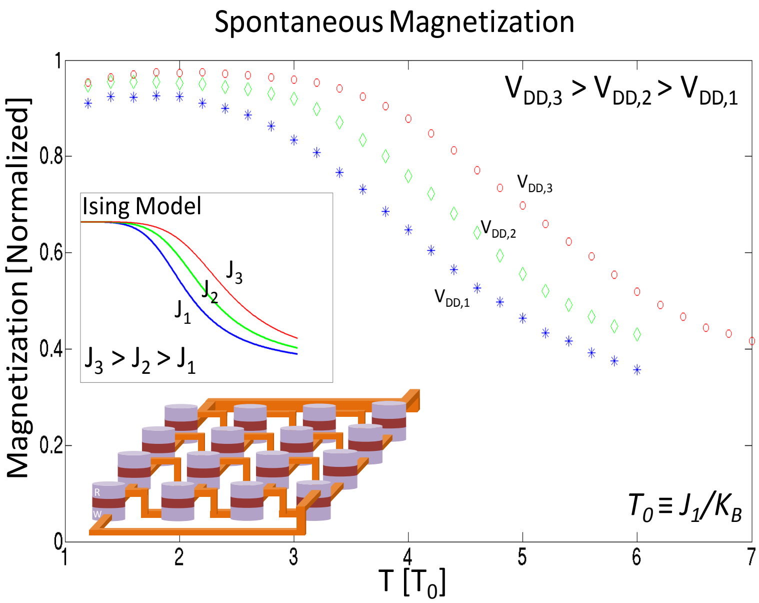

Figure 3: Effective magnetization in a network of Transynapses. Changing V_{DD} applied to each Transynapse can control the strength of the interaction between them somewhat like the J-coupling of Ising spins. Similar to the work of Ansager in describing T_c, spontaneous magnetization curves shift linearly as a function of the strength of the interactions. Higher V_{DD} makes the network to retain its magnetization at higher temepratures effectively raising the effective T_c in a linear fashion.

plot based on the Ising model illustrating the linear shift of spontaneous magnetization curves. Note also that for Figure 4 of the main paper, the data for magnetization has been smoothed out using the MATLAB function "smooth" before taking the derivative.

References

[1] K. Miura et al., CoFeB/MgO based perpendicular magnetic tunnel junctions with stepped structure for symmetrizing different retention times of "0" and "1" information. *Symposium on VLSI Technology Digest*, 11B-3, (2011).

[2] J. Z. Sun et al. , Effect of subvolume excitation and spin-torque efficiency on magnetic switching, *Physical Review Letter*, vol. **84,** 064413 (2011).

[3] M. Gajek et al. , Spin torque switching of 20nm magnetic tunnel junctions with perpendicular anisotropy. *Applied Physics Letters*, vol. **100,** pp. 132408.1-3 (2012).
